# Dynamic of Livestock-Associated Methicillin-Resistant *Staphylococcus aureus* CC398 in Pig Farm Households: A Pilot Study

**DOI:** 10.1371/journal.pone.0065512

**Published:** 2013-05-31

**Authors:** Cristina Garcia-Graells, Brigitte A. G. L. van Cleef, Jesper Larsen, Olivier Denis, Robert Skov, Andreas Voss

**Affiliations:** 1 Laboratoire de Référence MRSA-Staphylocoques, Department of Microbiology, Erasme Hospital-Université Libre de Bruxelles, Brussels, Belgium; 2 Laboratory of Medical Microbiology and Infection Prevention, St. Elisabeth Hospital, Tilburg, The Netherlands; 3 Center for Infectious Diseases Control, National Institute of Public Health and the Environment, Bilthoven, The Netherlands; 4 Microbiology and Infection Control, Statens Serum Institute, Copenhagen, Denmark; 5 Radboud University Nijmegen Medical Centre, Department of Medical Microbiology, Nijmegen, The Netherlands; 6 Department of Medical Microbiology and Infectious Diseases, Canisius-Wilhelmina Hospital, Nijmegen, The Netherlands; University of Iowa, United States of America

## Abstract

The aim of this study was to determine the long-term carriage rates and transmission dynamics of methicillin-resistant *Staphylococcus aureus* (MRSA) in pig farmers and their household members. During a 6-month period in 2009–2010, 4 pig farms in Denmark, Belgium, and the Netherlands, respectively, were studied for the presence of MRSA. The proportion of persistent carriers was significantly higher among farmers than among household members (87% vs. 11%) and significantly higher in household members from Belgium compared to those from Denmark and the Netherlands (29% vs. 0% vs. 6%). Determinant analysis of MRSA carriage revealed that pig contact was the most important determinant for MRSA carriage among household members and that the increased MRSA carriage rate observed among household members from Belgium is linked to country-specific differences in pig exposure.

These findings demonstrated that even in pig farms with very high carriage rates of MRSA both in livestock and farmers, the risk for household members to acquire MRSA is limited and still depends strongly on pig exposure. By restricting access to the stables and exposure to pigs, MRSA acquisition by household members could be greatly reduced.

## Introduction

Methicillin-resistant *Staphylococcus aureus* (MRSA) is a threat to public health worldwide. Next to the well-known hospital-associated and community-associated clones, another specific clone unrelated to the aforementioned has been discovered, which originates from an extensive reservoir in food-producing animals: livestock-associated (LA-) MRSA. This clone belongs typically to multi-locus sequence type (ST) 398 and closely related STs within clonal complex (CC) 398, lacks Panton-Valentine leukocidin (PVL), and is resistant to tetracycline. The presence of LA-MRSA in pigs and pig farmers was first described in France [1] and evidence of pig-to-farmer transmission of this clone was subsequently observed in the Netherlands [2]. Several surveys in livestock in Europe confirmed a high prevalence of LA-MRSA in pigs [3], [4] and in livestock farmers (up to 30%) [5], [6], [7], but lower carriage rates in people living on farms but with limited direct contact to food-producing animals (2% to 16%) [5], [8], [9].

Moreover, previous studies have shown that LA-MRSA carriage was directly related to the intensity of livestock exposure [7], [8], [9]. Nasal carriage seems to be persistent for farmers continuously exposed to colonized livestock [10] or transient for people with sporadic or indirect contact [11]. In addition, secondary transmission and spread amongst humans has so far been studied in healthcare settings only and seems to be limited compared to other MRSA clones [12], [13]. To date, little is known about the potential transmission routes amongst humans living in farm settings, the original environment of LA-MRSA. Some studies have suggested that carriage in household members could depend on the presence of positive livestock farmers [8] and that the contaminated farm environment could contribute to the transmission as well [10], [14]. However, these studies were limited to a short period of time and the in-house farm environment was not studied.

Therefore, the aim of this longitudinal epidemiological study was to determine the long-term carriage rates and transmission dynamics of LA-MRSA in pig farmers and their household members.

## Materials and Methods

### Study design

We conducted a 6-month longitudinal study (8 sampling moments) of MRSA carriage among farmers and their household members living on pig farms with LA-MRSA in Belgium, Denmark, and the Netherlands during 2009–2010. This study was developed as a tool for potential larger future investigations and therefore included only a small sample size per country. In each country, 4 pig farms were selected based on the following criteria: presence of LA-MRSA in the pig herd, which was detected in previous national screening studies in which ten randomly selected animals per age group were nasally swabbed to determine the presence of MRSA CC398; farrow-to-finish production system; and ≥3 members of the farmer's household. All household members were asked to participate in the study after receiving full information from the investigators. The study protocol was approved by the Ethics Committee of the Erasme Hospital-Université Libre de Bruxelles in Belgium (protocol no. P2009/203), the Danish National Committee on Biomedical Research Ethics (protocol no. H-4-2009-112) and the Danish Data Protection Agency (protocol no. 2009-54-0821), and the Medical Ethical Committee of the St. Elisabeth hospital in the Netherlands (protocol no. 0933). The volunteers signed an informed consent form and were asked to agree to nasal swabbing and to answer standard questionnaires at each sampling moment. In cases where children were under the age of 18 years, written consent was obtained from their parents. Swabs were taken before and during pig exposure by the investigators (during exposure: midday samples) and by the volunteers themselves (before exposure: morning samples) using the Venturi Transystem (Copan Innovation, Brescia, Italy). Samples from the house environment (farmer's favorite dog or cat, farmer's favorite chair, outside door handles, and TV remote control) were taken by the investigators using Sodibox wet wipe cloths (Sodibox, Nevez, France). The rate of sample collection was calculated considering available human and environmental samples at the time of the visit, some samples were missed for several reasons, among them: absence of households in the moment of the visit or impossibility to access into the farmer's house.

### Microbiological analysis

MRSA from individual samples was detected using standard methods [6]. Briefly, swabs were inoculated in a brain heart infusion enrichment broth containing 7.5% NaCl and incubated for 24 h at 35°C. Subsequently, 10 µl of a 0.5× McFarland suspension was inoculated onto chromID MRSA agar plates (bioMérieux, Marcy l'Etoile, France) and incubated for 24 h at 35°C. One suspected staphylococcal colony was selected from each plate and purified twice on Columbia agar with 5% sheep blood (Bio-Rad, Belgium) for further identification.

Species identification, presence of the *lukF*-*lukS* genes encoding PVL, and resistance to methicillin were confirmed by a triplex PCR assay [15]. Isolates (*N* = 100) from the first and last sampling moment at which MRSA was isolated from each volunteer/environmental site, respectively, was characterized by *spa* typing using the Ridom Staph Type standard protocol (http://www.ridom.com) and the Ridom SpaServer (http://spa.ridom.de/index.shtml), SCC*mec* typing [16], and antimicrobial susceptibility testing (spectinomycin, gentamicin, kanamycin, tobramycin, rifampin, trimethoprim-sulfamethoxazole, clindamycin, erythromycin, linezolid, chloramphenicol, mupirocin, ciprofloxacin, minocycline, tetracycline, and fusidic acid) using Neo-Sensitabs (Rosco, Taastrup, Denmark) in accordance with the Clinical Laboratory Standards Institute guidelines (CLSI 2011), as described elsewhere [17]. Multidrug resistance (MDR) was defined as resistance to ≥4 non-β-lactam antimicrobial classes. MLST [18] was performed on MRSA isolates representing each *spa* type.

The genetic relatedness of a subset of these isolates (*N* = 36) representing one farm per country was further assessed by pulsed-field gel electrophoresis (PFGE) using *Cfr*9I (Fermentas Gmbh, Germany) as previously described [19]. PFGE patterns were analysed using Bionumerics version 6.5 (Applied Maths, Kortrijk, Belgium) according to previously described criteria [20].

### Epidemiological data

Demographic data (gender, age, occupation, status in the family), farm- and animal-related variables (exposure to pigs, cattle, poultry, horses, and pets, handling antimicrobial drugs to pigs, use of hygiene/protective measures, and occupational activities), life style determinants (eating preferences, exposure to raw meat, smoking, contact sports, travel), and medical history (exposure to health care facilities, antibiotic usage) were collected for each volunteer at each sampling moment.

### Definitions

Volunteers were categorized into individuals exposed to pigs >30 hours per week and individuals exposed to pigs ≤30 hours per week, on average (termed farmers and household members, respectively), and were assigned to 1 of 3 groups with regard to MRSA carriage: persistent carriers (100% of the cultures were positive for MRSA), non-carriers (100% of the cultures were negative for MRSA), and intermittent carriers (all other volunteers).

### Statistical analysis

The data were analyzed using SAS software version 9.2 (SAS Institute Inc., Cary, North Carolina, USA). Comparison of proportions was done with Chi-square tests, or Fisher's exact tests when expected cell counts were below 5. Determinants for MRSA carriage in household members were stratified per country. All tests were 2-sided, and the significance level was set at *P* = 0.05.

## Results

### MRSA carriage in the study population

A total of 60 persons (20 per country) participated in the study, including 15 farmers and 45 household members (Table 1). Altogether, 453 midday samples (both farmers and household members, sample collection rate 95% [453/480]), 69 morning samples (farmers only, sample collection rate 71% [69/96]), and 357 environmental samples (sample collection rate 93% [357/384]) were analyzed. The proportion of persistent carriers was significantly higher among farmers than among household members (87% vs. 11%; Fisher's exact *P*<0.0001, Table 2) and did not vary between countries (farmers from Belgium, 100%; Denmark, 80%; the Netherlands, 75%; Fisher's exact *P* = 0.49). The majority (87% [60/69]) of morning samples from farmers were positive for MRSA.

**Table 1:** Methicillin-resistant *Staphylococcus aureus* (MRSA) carriage among 15 farmers and 45 household members.

Category, country	No. of non-carriers (%)	No. of intermittent MRSA carriers (%)	No. of persistent MRSA carriers (%)
Farmers			
Belgium	0 (0)	0 (0)	6 (100)
Denmark	0 (0)	1 (20)	4 (80)
The Netherlands	0 (0)	1 (25)	3 (75)
Total	0 (0)	2 (13)	13 (87)
Household members			
Belgium	2 (14)	8 (57)	4 (29)
Denmark	14 (93)	1 (7)	0 (0)
The Netherlands	13 (81)	2 (13)	1 (6)
Total	29 (64)	11 (24)	5 (11)

**Table 2:** Isolation rates of methicillin-resistant *Staphylococcus aureus* in 357 environmental samples.

Origin	No. of positive samples (%)			
	Belgium	Denmark	The Netherlands	Total
Dog or cat	11 (48)	9 (29)	2 (6)	22 (26)
Chair	9 (29)	10 (31)	5 (16)	24 (25)
Outside door handle	11 (46)	2 (6)	0 (0)	13 (15)
Television remote control	12 (50)	7 (22)	0 (0)	19 (22)
Total	43 (42)	28 (22)	7 (5)	78 (22)

The proportions of both intermittent and persistent MRSA carriers were significantly higher in household members from Belgium compared to Denmark and the Netherlands (intermittent: 57% vs.7% vs. 13% [Fisher's exact *P* = 0.004] and persistent: 29% vs. 0% vs. 6% [Fishers's exact *P* = 0.03]).

### Environmental samples

The isolation rates of MRSA in the environmental samples are shown in Table 2. The overall isolation rate from environmental samples was 22% (78/357), with important geographic variations (Belgium 42%; Denmark 22%; the Netherlands, 5%; BE vs DK *P* = 0.0004; BE vs NL *P*<0.0001; NL vs DK *P* = 0.0001).

Our study did not show a positive association between environmental samples and MRSA carriage in household members (100% (9/9) of Belgian household members with MRSA in environmental samples were MRSA positive during the study, compared to 60% (3/5) of Belgian household members from MRSA-negative environments, *P* = 0.11; for Danish household members these numbers were 10% (1/10), 0% (0/5), *P* = 1.00; and for Dutch household members 23% (3/13), 0% (0/3), *P* = 1.00).

### Molecular and phenotypic characterization

**Figure 1:**
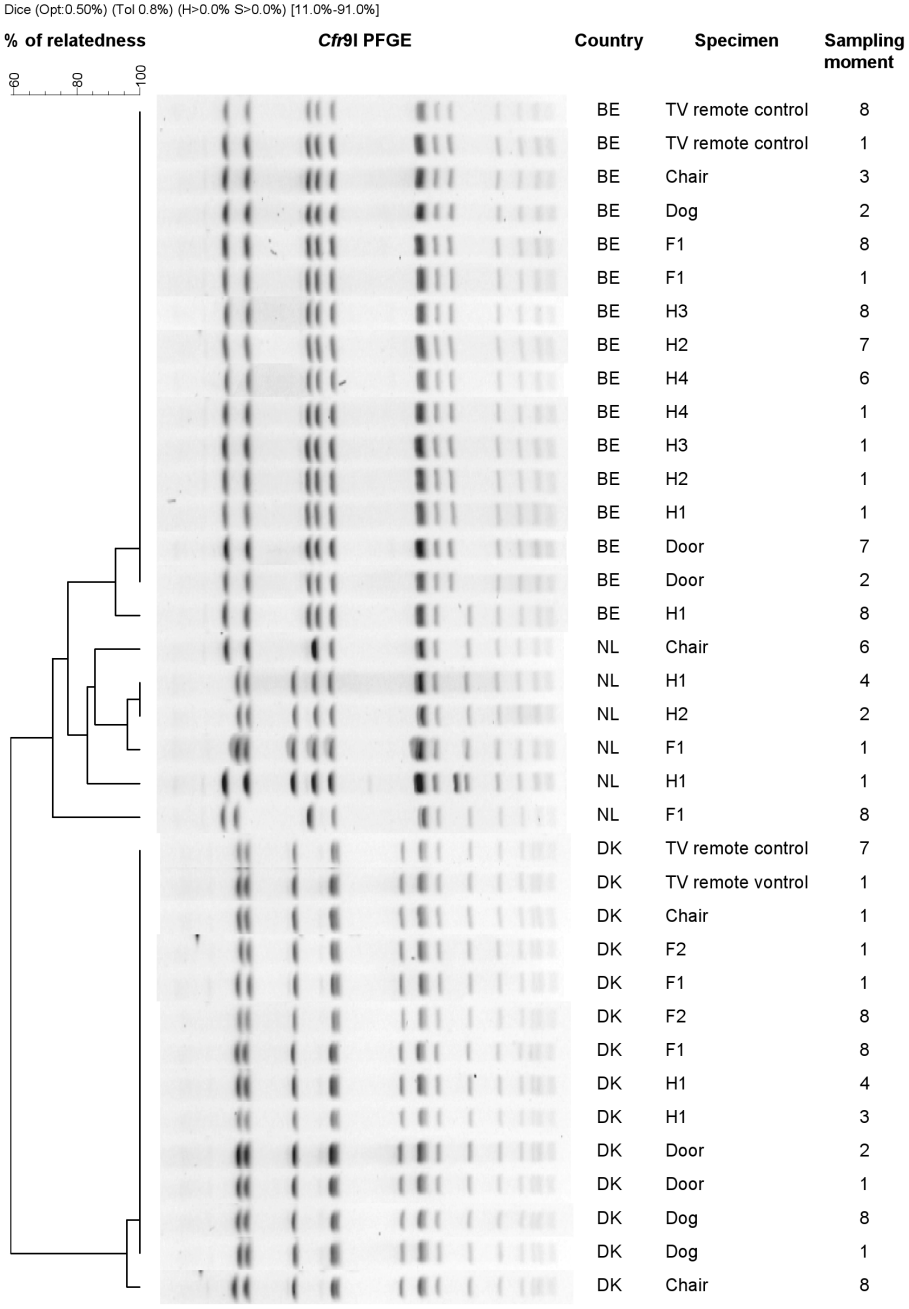
PFGE patterns of MRSA isolates (N = 36) from a single farm per country (Belgium n = 16, Denmark n = 14, Netherlands n = 6).

All 100 isolates subjected to molecular genotyping and antimicrobial susceptibility testing had characteristics that were compatible with LA-MRSA CC398: they displayed closely related *spa* types t011, t034, t0108, t1451, t2370, and t6017 and belonged to ST398 within CC398; they lacked the *lukF-lukS* genes encoding PVL; they carried SCC*mec* type V (92%) or IV (8%); and they were resistant to tetracycline (100%). In addition, 19% were MDR. Isolates recovered from farmers, household members, and environmental samples from each farm were highly homogeneous in terms of *spa* typing, SCC*mec* typing, and antimicrobial susceptibility patterns (data not shown). Furthermore, isolates originating from the same farm had indistinguishable PFGE patterns (Figure 1).

### Determinants of MRSA carriage among household members

Pig contact rate (hours per week), exposure to pigs within the last 7 days, contact to sows, and handling antimicrobial drugs to pigs were significantly associated with MRSA carriage among household members (Table 3), whereas no associations were found for gender, age, status in the family, other occupations, eating preferences, exposure to raw meat, smoking, contact sports, travel, medical history, exposure to other animals (cattle, poultry, horses, and pets), use of hygiene/protective measures, presence of a farmer with MRSA in the household, and presence of MRSA in the household environment.

**Table 3:** Determinants for persistent and intermittent methicillin-resistant *Staphylococcus aureus* (MRSA) carriage among household members.

Determinant	Value	Belgium			Denmark			Netherlands		
		Total no.	No. of carriers (%)	*P*	Total no.	No. of carriers (%)	*P*	Total no.	No. of carriers (%)	*P*
Total		14	12 (86)		15	1 (7)		16	3 (19)	
Pig exposure time (hours per week)	10–30	4	4 (100)	1,00	1	0 (0)	1,00	2	2 (100)	**0,03**
	<10	10	8 (80)		14	1 (7)		14	1 (7)	
Exposure to pigs within last 7 days	Yes	11	11 (100)	**0,03**	3	0 (0)	1,00	6	3 (50)	**0,04**
	No	3	1 (33)		12	1 (8)		10	0 (0)	
Contact to sows	Yes	9	9 (100)	0,11	3	0 (0)	1,00	6	3 (50)	**0,04**
	No	5	3 (60)		12	1 (8)		10	0 (0)	
Handling antimicrobial drugs to pigs	Yes	1	1 (100)	1,00	1	0 (0)	1,00	2	2 (100)	**0,03**
	No	13	11 (85)		13	1 (8)		14	1 (7)	

Notes: *P*, Fisher's Exact *P* value. *P* values in bold indicate significant differences.

**Figure 2:**
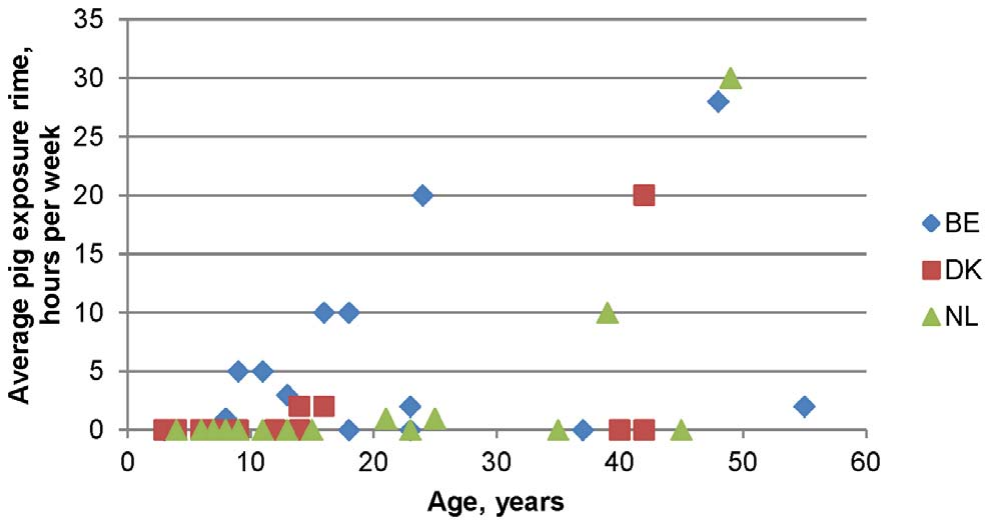
Association between country, age, and average pig exposure time among household members in each country. BE, Belgium; DK, Denmark; NL, the Netherlands.

The association between country, age, and average pig exposure time among household members in each country is illustrated in Figure 2. In general, household members from Belgium were more exposed to pigs and at an earlier age compared to household members from the Netherlands and Denmark. These findings suggest that the increased MRSA carriage rate observed among household members from Belgium is linked to country-specific differences in pig exposure.

## Discussion

In this study, we found that 87% of pig farmers were persistent LA-MRSA nasal carriers for a period of at least 6 months. Moreover, the majority of the pig farmers tested MRSA positive before exposure to pigs, which is consistent with persistent carriage rather than re-acquisition and loss on a daily basis. This finding supports that farmers can be a source of household transmission. However, presence of a farmer with MRSA could not be associated with household transmission, since all farmers were MRSA positive during the study.

The carriage rate found in this study is much higher than previously reported on positive pig farms (49% of farmers and 6% of household members) [5], veal farms (positive and negative farms combined: 38% of farmers and 16% of household members) [8], and in field workers visiting MRSA positive pig and veal farms (48% of field workers) [11] in the Netherlands. In addition, lower rates were found in Belgium as well (37.8% of farmers, co-workers and households in positive and negative farms combined) [6]. This could be the result either of a rising MRSA prevalence in people over time, or, more likely, due to the limited number of farms per country included in this study. A very remarkable finding was the large difference in LA-MRSA carriage rate among household members of the different countries. In Denmark and the Netherlands, the carriage rate, defined as intermittent and persistent carriers together, ranged between 7–19%, which is comparable to the MRSA nasal carriage rates found in family members of Dutch veal calf farmers (16%) [8], but much higher than the 0.2% reported in the Netherlands in people without any livestock contact [21]. In Belgium, a dramatically high carriage rate was found among household members (86%), which was comparable to that of the farmers. This can be explained by our finding that household members in Belgium were more exposed to pigs and at an earlier age compared to household members from the Netherlands and Denmark where exposure to pigs was largely restricted to farmers. As expected, all MRSA isolates shared typical characteristics of LA-MRSA in terms of *spa* typing and MLST, lack of the *lukF-lukS* genes encoding PVL, SCC*mec* typing, and antimicrobial susceptibility patterns as previously reported [6], [8], [9], [17] A novel finding was the frequent isolation of LA-MRSA in Belgian farm house environments (42%), which can be a reflection of the higher LA-MRSA carriage rate among Belgian household members. Although it has been suggested that the environment might play a role in LA-MRSA transmission amongst family members, our study did not show a positive association between environmental samples and MRSA carriage in household members (100% (9/9) of Belgian household members with MRSA in environmental samples were MRSA positive during the study, compared to 60% (3/5) of Belgian household members from MRSA-negative environments, Fisher's exact *P* = 0.11; for Danish household members these numbers were 10% (1/10), 0% (0/5), *P* = 1.00; and for Dutch household members 23% (3/13), 0% (0/3), *P* = 1.00). However, the high rates found in companion animals, particularly in Belgium, have to be interpreted with caution since the role of pets as potential vectors and/or reservoirs of LA-MRSA is still not clear and needs future research. Notably, the finding that exposure to a persistent carrier (farmer) did not imply a risk for spread to household members confirms that human-to-human transmission of this clone seems to be very limited, as previously reported [12], [13], [22].

Our results are of interest when developing strategies for preventing spread of LA-MRSA to household members of pig farmers. By restricting access to the stables and exposure to pigs, the risk of LA-MRSA acquisition by household members could be greatly reduced. However, this needs further investigation and confirmation by future studies.

In conclusion, we have demonstrated that even in pig farms with a very high carriage rates of MRSA in both livestock and pig farmers, the risk for household members to acquire MRSA is limited and depends strongly on pig exposure.
